# Molecular diagnosis of urogenital schistosomiasis in pre-school children, school-aged children and women of reproductive age at community level in central Senegal

**DOI:** 10.1186/s13071-023-05671-x

**Published:** 2023-01-31

**Authors:** Doudou Sow, Khadime Sylla, Ndeye Marème Dieng, Bruno Senghor, Papa Mouhamadou Gaye, Cheikh B. Fall, Ndiaw Goumballa, Aldiouma Diallo, Jean Louis A. Ndiaye, Philippe Parola, Cheikh Sokhna, Souleymane Doucouré, Babacar Faye

**Affiliations:** 1 Service de Parasitologie-Mycologie, UFR Sciences de la Santé, Université Gaston Berger, Saint-Louis, Sénégal; 2 Service de Parasitologie-Mycologie, FMPO, Université Cheikh Anta Diop, Dakar, Sénégal; 3 Unité VITROME Dakar, Institut de Recherche pour le Développement, Dakar, Sénégal; 4 Service de Parasitologie-Mycologie, UFR Sciences de la Santé, Université Iba Der Thiam, Thies, Sénégal; 5 Unité VITROME, IHU Méditérannée Infection, Marseille, France

**Keywords:** Urogenital schistosomiasis, Real-time PCR, Pre-school children and school-aged children, Women of reproductive age

## Abstract

**Background:**

Urogenital schistosomiasis is a major public health concern in sub-Saharan Africa. In Senegal, the disease is endemic in all regions of the country. Recently, WHO strongly recommended including pre-school children and women of reproductive age during a mass drug administration campaign. It is important to describe the burden of the disease in these group at risk using innovative diagnostic tools. This study aimed to assess the use of real-time PCR in the detection of schistosomiasis cases at the community level in a seasonal transmission area.

**Methods:**

A cross-sectional survey was carried out in Niakhar located in the centre of Senegal. Pre-schoolchildren, school-aged children and female adolescents and adults were invited to participate in the study in April 2018. Urine samples were collected and examined using Hemastix reagent strips, filtration technique and real-time PCR. *Schistosoma haematobium* was detected, identified by targeting the Dra1 gene. The prevalence of urogenital schistosomiasis was determined for each group and the performance of the real-time PCR was compared with the conventional techniques.

**Results:**

A total of 428 participants were enrolled in this study including 87 (20.4%) pre-school children (1–5 years), 262 (61.3%) school-aged children between (5–14 years), 17 (3.9%) adolescents (15–17 years) and 62 (14.4%) female adults. The comparison of the diagnostic techniques has shown that the prevalence of urogenital schistosomiasis is higher using molecular technique (34.6%) compared to microscopy (20.3%). The percentage rate of haematuria using Hemastix was 23.1%. School-aged children between 5 and 14 years old were the most affected with 29.0% and 43.1% under microscopy and RT-PCR, respectively. In female participants, microscopic prevalence decreases with age, from 21.4% in school-aged children to 17.6% in adolescents and 9.7% in adults. There was good correlation between the number of eggs per 10 ml and the cycle threshold range.

**Conclusion:**

These results show the importance of using molecular tools in the surveillance of schistosomiasis particularly in pre-school children and women of reproductive age.

**Graphical Abstract:**

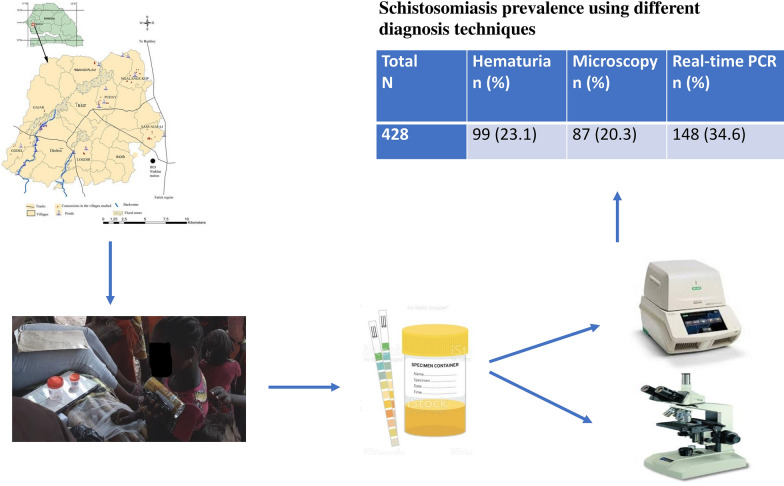

## Introduction

Schistosomiasis is one of the most endemic and important neglected tropical diseases in Africa, South America and Asia. Approximately 779 million people are at risk of developing the disease [1]. In 2020, it was estimated that 241.3 million people needed praziquantel treatment for preventive chemotherapy [2]. Despite the control strategies implemented for several years, schistosomiasis prevalence is still high in many endemic regions, particularly in Africa. Therefore, the WHO strongly recommends including pre-school-aged children (PSC), pregnant women, women of reproductive age and adults during praziquantel mass drug administration campaigns [1].

*Schistosoma haematobium* and *Schistosoma mansoni*, responsible for urogenital and intestinal schistosomiasis, respectively, are the most common species circulating in sub-Saharan Africa [3]. Both species are known to cause chronic diseases in humans, particularly *Schistosoma haematobium*. Indeed, infection by this species can lead to bladder or cervical cancer in women after chronic inflammation because the eggs become trapped in tissues [4]. Therefore, it is particularly important to have sensitive diagnostic tools able to detect the presence of parasites even at low level intensity. Currently, microscopic methods including the filtration technique are the main tools used in the field for the diagnosis of urogenital schistosomiasis. However, it is well known that this method lacks sensitivity in persons with low-level parasite density or chronic infection because of the small numbers of eggs excreted in urine samples. Point-of-care circulating cathodic antigen (POC-CCA) is an immunological test that has been proposed for the detection of schistosomiasis cases. Unfortunately, this method is more effective at detecting *Schistosoma mansoni* than *Schistosoma haematobium* [5]. For several years, molecular methods including standard and real-time PCR have been evaluated in the field for detection of schistosomiasis cases [6–10]. These techniques have been shown to be more sensitive than other methods for detecting low-level *Schistosoma* infections. Therefore, it would be an interesting alternative method for the surveillance of cases in endemic areas to target the elimination of schistosomiasis.

In Senegal, urogenital schistosomiasis is endemic in all regions of the country. In the northern part, transmission occurs all year. The prevalence is very high in these regions, reaching 82% in school-aged children in many villages [11, 12]. However, transmission of the disease is seasonal in the centre of the country and the prevalence rate was at 56.7% in a previous survey in Niakhar, region of Fatick [13]. In this particular area, the implementation and the sustainability of control strategies could allow elimination of schistosomiasis as a public health problem in this part of the country. To achieve this objective, it will be relevant to include pre-school-aged children and females of reproductive age in the preventive chemotherapy campaign. This last group is particularly important to follow to prevent the risk of female genital schistosomiasis, which can cause cervical cancer. This study was carried out to describe the molecular prevalence of schistosomiasis in school-aged children and other groups at risk, pre-school-aged children and women of reproductive age, in a seasonal transmission area.

## Methods

### Study area

This study was performed in Niakhar health district (Fatick region), located in the central part of Senegal, 135 km from the capital, Dakar. The villages of Niakhar have been under health and demographic surveillance for several decades by the Institute of Research and Development (IRD). Climate is Sudano-Sahelian with an annual rainfall of approximately 506 mm. Niakhar health district is composed by 30 villages with a total population of 43,000 inhabitants [14]. Previous environmental surveys have shown the number of water sources present in the area (wells, ponds, supply taps, backwaters, etc.) and the important number of villages with no access to tap water [15]. The district of Niakhar is endemic to urogenital schistosomiasis [16]. The Niakhar area was chosen because of the availability of schistosomiasis baseline data collected during a previous survey [13, 16, 17]. The communities have received a round of praziquantel mass treatment each year since 2016 for school-age children.

### Study design and selection of participants

**Fig. 1:**
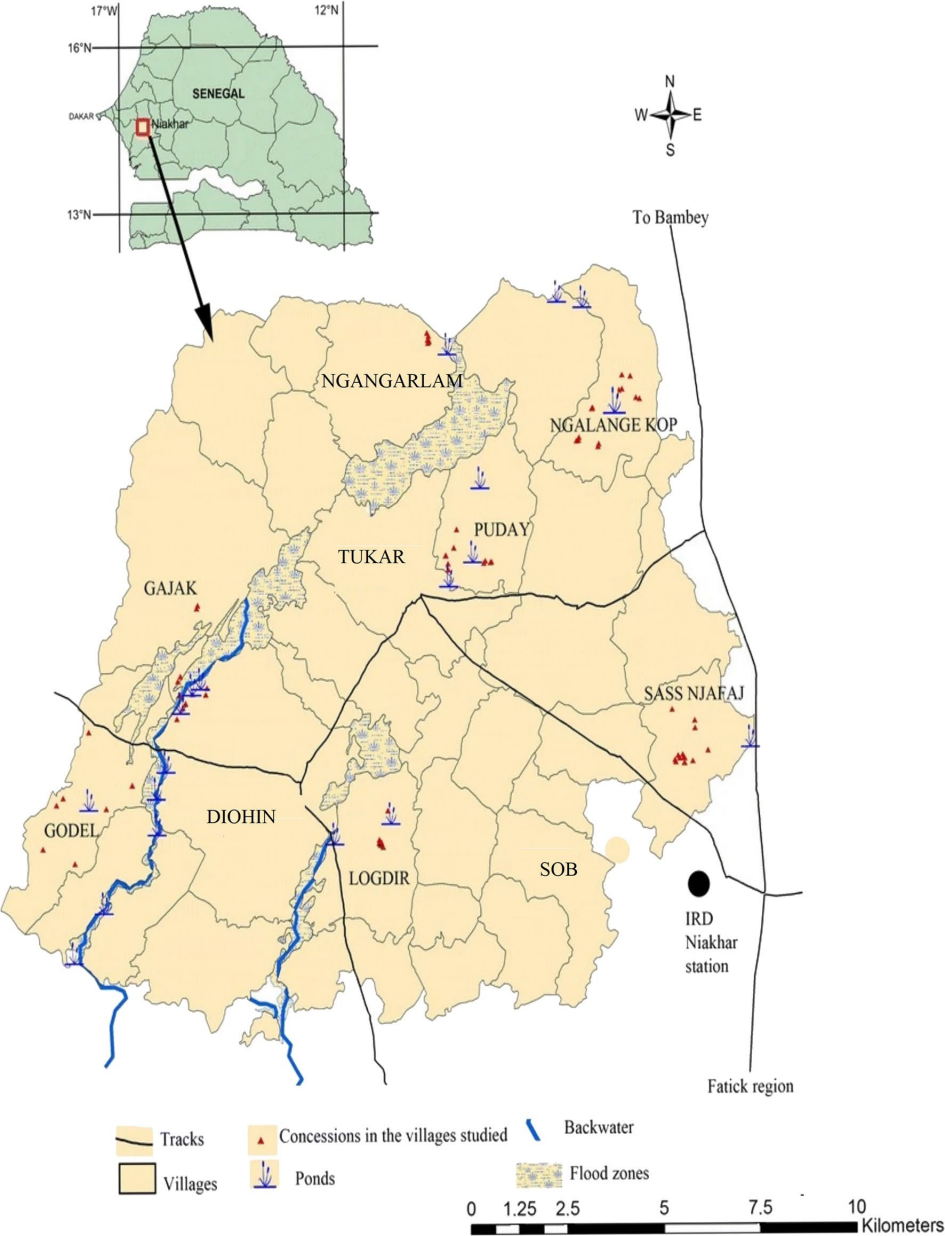
Map of the study area of Niakhar showing the 10 study villages [13]

A cross-sectional survey was carried out from 6 to 14 April 2018 in 10 villages in Niakhar. Study sites was selected based on urogenital schistosomiasis endemicity as shown by previous studies [13, 17]. The selected villages are represented in Fig. 1. The sample size was calculated according to the size of the total population of each village. Based on a prevalence estimated at 10%, a confidence level of 95% and alpha risk of 5%, the number of individuals to be included in the study was estimated at 428. The total number was weighted according to the size of each village.

Children, including pre-school (1–4 years) and school-age children (5-14 years), and women of reproductive age (15-49 years) living in the study area were included in the study. All the individuals who had taken praziquantel treatment 6 months before the survey were excluded during the screening process.

### Sample collection and microscopic examination

In each village, urine samples were collected in a single day between 10:30 a.m. and 2:00 p.m. in appropriate sealed, labelled, clean pots. After macroscopic haematuria examination, microscopic haematuria was checked using reagent sticks (Hemastix, BAYER^®^). The samples were then stored at 4 °C in icepacks and transported to the laboratory. Microscopic examination was done using urine filtration technique following the WHO standard procedure [18]. A 10-ml volume of urine was filtered through polycarbonate filters. The filters were then read after adding a drop of Lugol staining using microscopy at 100× and 400× magnification, and the result was standardized to number of eggs/10 ml urine. The infection intensity was classified as light (< 50 eggs/10 ml urine) or heavy (≥ 50 eggs/10 ml urine) following the WHO recommendations [1].

### DNA extraction and PCR assays

DNA was extracted from urine samples using the E.Z.N.A.® Tissue DNA Kit (Omega Bio-tek, USA). The extraction, purification and elution of the DNA were done following the manufacturer’s recommendations.

The real-time PCR assay was done targeting the *Schistosoma haematobium* Dra1 gene as previously described [19]. Primers and probe sequences were as follows: Sh-FW: 5′-GATCTCACCTATCAGACGAAAC-3′; Sh-RV: 5′-TCACAACGATACGACCAAC-3′; Sh-probe: 5′-FAM-TGTTGGTGGAAGTGCCTGTTTCGCAA-TAMRA-3′.

The real-time reaction mixture was prepared using 10 µl ROCHE^®^ Master mix, 0.5 µl of each primer and probe, 3.5 µl nuclease-free water and 5 µl extracted template DNA giving a total volume of 20 µl. Real-time PCR assay was performed on a CFX96™ machine (BIO-RAD, Life Science, Marnes-la-Coquette, France). The cycling conditions were as follows: 5 min denaturation at 95 °C followed by 39 cycles of 30 s denaturation at 95 °C and 60 s hybridization at 60 °C.

### Data analysis

Data were first collected using questionnaires and then entered into Excel software. The statistical analysis was done using the TM R2.15.0 software (R Foundation for Statistical Computing, Vienna, Austria). Quantitative variables were described in terms of means while qualitative data were presented in number and percentage of data provided. The Chi-square or Fischer test was used to perform statistical comparisons depending on the condition of applicability. A test result of *P* < 0.05 was considered significant.

To assess the performance of real-time PCR, the sensitivity, specificity and positive and negative predictive value were calculated. The kappa coefficient to assess the level of correlation between the techniques was also calculated. The kappa measure was interpreted as follows: *k* = 0.21–0.40, acceptable correlation; *k* = 0.41–0.60, moderate correlation; *k* = 0.61–0.80, significant correlation; *k* = 0.81–1, almost perfect correlation.

The Youden index provides an overall measure of the statistical performance of a test. The value is between 0 and 1. The interpretation is as follows: 0 = inefficient test; 1 = perfect test.

## Results

### Sociodemographic characteristics of study population

Overall, 428 participants were enrolled in this study including 87 (20.4%) pre-school children (1–4 years), 262 (61.3%) participants aged between 5 to 14 years old, 17 (3.9%) adolescents aged 15 to 17 years and 62 (14.4%) female adults. Among the pre-school- and school-aged children, there were 192 (44.8%) boys and 157 (36.6%) girls. The adolescents and adults were female and considered as women of reproductive age. All these participants were recruited from 10 villages (Table 1).

**Table 1:** Schistosomiasis prevalence using different diagnostic techniques

Parameters	*N*	Haematuria		Microscopy				Real-time PCR			
		*n* (%)	*P* value	*n* (%)	*P* value	Eggs	*P* value	*n* (%)	*P* value	CT Means ± SD	*P* value
						Means ± SD					
Total	428	99 (23.1)		87 (20.3)		264 ± 344		148 (34.6)		31 ± 6	
Age			< 0.001*		< 0.001*		0.300		< 0.001*		< 0.001*
< 5 years	87	3 (3.4)		2 (2.3)		15 ± 19		11 (12.6)		35 ± 4	
5–14 years	262	75 (29.8)		76 (29.0)		284 ± 348		113 (43.1)		30 ± 6	
15–17 years	17	9 (35.3)		3 (17.6)		337 ± 5757		5 (29.4)		32 ± 6	
18–49 years	62	12 (19.4)		6 (9.7)		54 ± 29		19 (30.6* )		33 ± 5	
Gender			0.055		0.001*		0.023*		0.146		0.001*
Boys	192	54 (28.1)		54 (28.1)		342 ± 385		76 (39.6)		30 ± 6	
Girls	157	27 (17.2)		24 (15.3)		131 ± 163		48 (30.4)		32 ± 5	
Adult females	79	18 (22.8)		9 (11.4)		148 ± 321		24 (30.8)		33 ± 5	
Villages			< 0.001*		< 0.001*		0.082		< 0.001*		< 0.001*
Diohin	77	18 (23.4)		16 (20.8)		326 ± 397		27 (35.1)		29 ± 5	
Gajak	94	29 (30.9)		25 (26.6)		181 ± 225		33 (35.1)		27 ± 5	
Godel	29	10 (34.5)		8 (27.6)		206 ± 214		17 (58.6)		28 ± 3	
Logdir	48	3 (6.3)		3 (6.3)		34 ± 28		5 (10.4)		32 ± 3	
Ngalange Kop	26	0 (0)		0 (0)				3 (11.5)		31 ± 6	
Ngangarlam	43	17 (39.5)		18 (41.9)		467 ± 448		25 (58.1)		27 ± 5	
Puday	27	15 (55.6)		14 (51.9)		198 ± 346		18 (66.7)		27 ± 4	
Sass NjafaJ	30	1 (3.3)		0 (0)				1 (3.3)		31	
Sob	47	4 (8.5)		3 (6.4)		101 ± 54		17 (36.2)		29 ± 2	
Tukar	7	2 (28.6)		0 (0)				2 (28.6)		31 ± 5	

^*^Statistically significant

### Prevalence and intensity of *Schistosoma haematobium* infection

Of the 428 participants tested, 99 (23.1%) were positive for haematuria. Microscopy revealed the prevalence of urinary schistosomiasis was 20.3% (87/428) in Niakhar area. Prevalence of heavy infections (> 50 eggs/10 ml) was 12.9% (55/428) while 32 (7.4%) had excreted < 49 eggs/10 ml. Prevalence and infection intensity according to age, gender and within villages are presented in Table 1. The highest microscopic prevalence of 29% (76/262) was observed in school-aged children and the lowest at 2.3% (2/87) was reported in pre-school children. In women of reproductive age, the microscopic prevalence rate was 17.6% in adolescents and 9.7% in adult females.

Microscopic prevalence varied from 0 in three villages (Ngalagne Kop, Sass Ndiafadji and Tukar) to 51.9% in Puday.

When considering the age groups, the Hemastix test detected more haematuria cases in adolescents (35.3%) and school-aged children (29.8%) compared to pre-school children and adult females with significant differences (*p* < 0.001) (Table 1). The microscopic prevalence of schistosomiasis was more important in these two age groups as well. The prevalence rate was 29% in school-aged children and 17.6% in adolescents.

Using the real-time PCR technique, a higher prevalence of 34.6% (148 of 428) was noted. The majority of positive cases have shown high DNA load with cycle threshold values (Ct values) < 30 (mean = 27.9). As observed with microscopy, the prevalence was higher in school-aged children (43.1%). However, real-time PCR detected more cases in adults (30.6%) compared to adolescents (29.4%). In preschool-aged children, the real-time PCR yielded a high prevalence rate (12.6%) compared to microscopy (2.3%). Boys were more affected than girls using both techniques (Table 2). In female participants, microscopic prevalence decreased with age. Indeed, the prevalence was 21.4% in school-aged children, 17.6% in adolescents and 9.7% in adults. However, molecular prevalence decreased between school-aged children and older but did not change much between adolescents and female adults (Tables 1, 2).

**Table 2:** Prevalence of schistosomiasis according to age and gender

Parameters		*N*	Hematuria		Micros				Real time PCR			
			*n* (%)	*P* value	*n* (%)	P value	Eggs	P value	*n* (%)	*P* value	CT Means ± SD	*P* value
							Means ± SD					
Total		428	99 (23.1)		87 (20.3)		264 ± 344		148 (34.6)		31 ± 6	
Age	Gender			0.517		0.139		-		0.082		0,010*
< 5 years	Boys	42	2 (4.8)		2 (4.8)		15 ± 19		8 (19.0)		32 ± 5	
	Girls	45	1 (2.2)		0 (0.0)		-		3 (6.7)		36 ± 2	
Age	Gender			0.045		0.019*		0.007*		0.405		0,094
5–14 years	Boys	150	52 (34.7)		52 (34.7)		355 ± 387		68 (45.3)		30 ± 6	
	Girls	112	26 (23.2)		24 (21.4)		131 ± 163		45 (40.2)		31 ± 5	

^*^Statistically significant

School-aged children between 5 and 14 years old were the most affected with 29.0% and 43.1% under microscopy and PCR, respectively. However, those < 5 years old were the least affected with prevalence of 2.3% on microscopy and 12.6% on PCR.

In total, two (2.3%) children under the age of 5 were affected by *Schistosoma* just like their mothers.

### Cycle threshold ranges and cut-offs

**Fig. 2:**
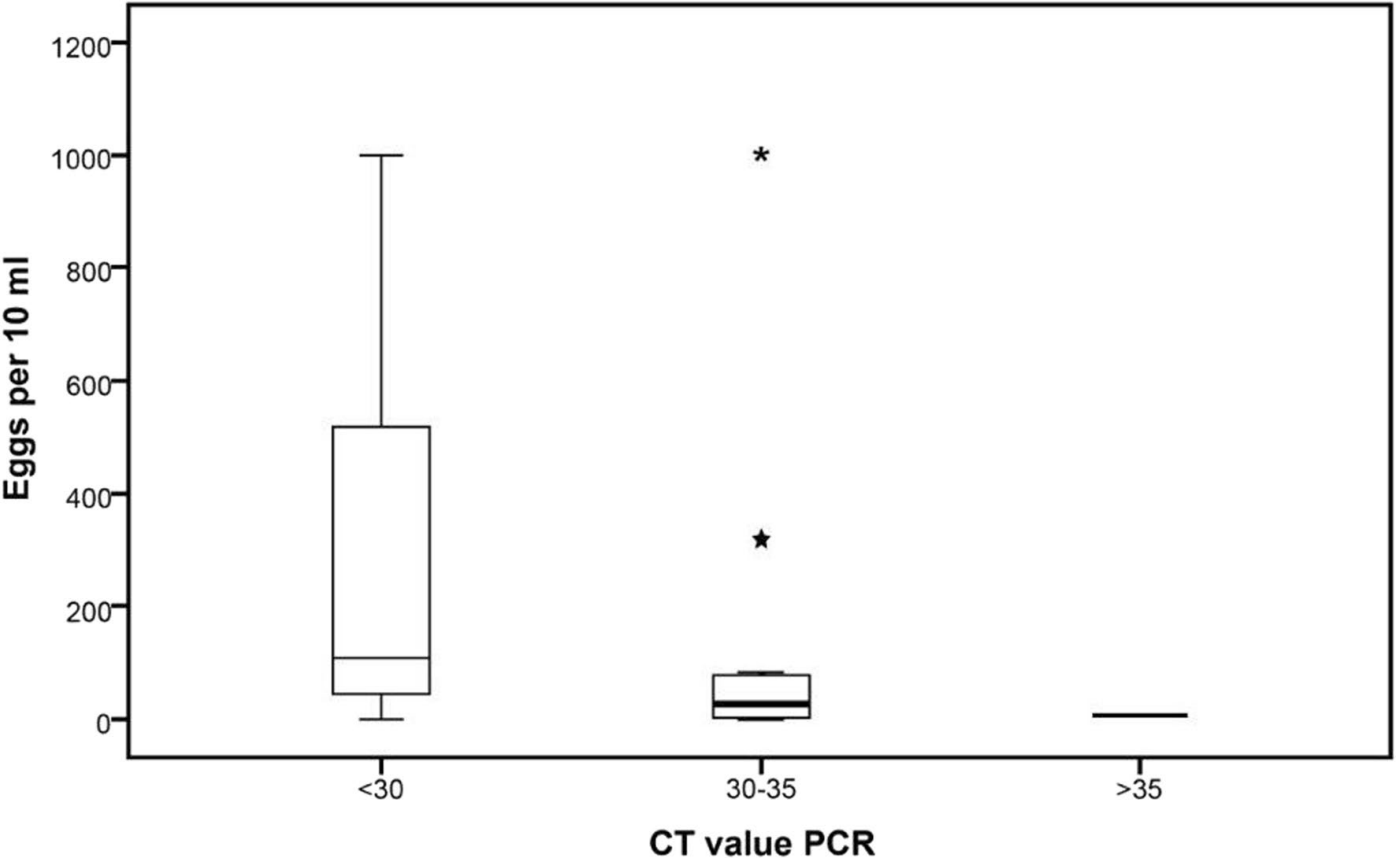
Comparison between the number of eggs per 10 ml and real-time PCR Ct value

Regarding the infection intensity, there was a good correlation between the number of eggs per 10 ml and cycle threshold range. The comparison was made on the 87 positive patients. The mean eggs per 10 ml (306.93 ± 358.2) was higher in the group with Ct value < 30 compared to the group with Ct value between 30 and 35 (mean eggs = 115.4 ± 241.5). The range of the number of eggs per 10 ml is larger in patients presenting a Ct value < 30 compared to patients with Ct values between 30 and 35 as presented in Fig. 2.

### Performance of real-time PCR compared to microscopy

The comparison of the techniques has shown that the prevalence of urogenital schistosomiasis is higher using molecular techniques (34.6%) compared to the microscopy (20.3%). Real-time PCR was particularly useful in detecting schistosomiasis cases in pre-school children and females of reproductive age. There was excellent sensitivity and specificity of real-time PCR technique and a good Youden coefficient (Table 3).

**Table 3:** Performance of the real-time PCR technique compared to microscopy

Real-time PCR	Microscopy								
	Positive	Negative	Total	Sensitivity [95% CI]	Specificity [95% CI]	VPP [95% CI]	VPN [95% CI]	Kappa	Youden
Positive	86	62	148	98.9 [97.8–99.9]	81.8 [78.2–85.5]	58.1 [54.4–62.8]	99.6 [99.1–100.2]	0,64	0,58
Negative	1	279	280						
Total	87	341	428						

## Discussion

This study has shown the important number of urogenital schistosomiasis cases in pre-school children and women of reproductive age when other methods were used compared to egg detection by microscopy. For school-aged children, the number of cases presenting haematuria was similar to the number positive to microscopy while the use of Hemastix strips gave more positive results in pre-school children and women of reproductive age compared to microscopy. This observation was reported by Ibironke et al. who demonstrated that the prevalence of urogenital schistosomiasis using Hemastix strip versus microscopy was quite similar in school-aged children [20]. However, another study showed that the detection of haematuria had low accuracy when the infection level was around 1–5 eggs per 10 ml urine [21]. This could explain why the correlation between these two techniques was interesting in our study in school-aged children as the mean number of eggs per 10 ml of urine was more important in this group (288 ± 348).

The Hemastix test has been described to be an interesting tool for epidemiological survey due to its high sensitivity and low cost. However, the specificity could be a limiting aspect when using it on a large scale.

In our study, the overall prevalence of urogenital schistosomiasis obtained by real-time PCR was higher (34%) than that by microscopy (20%). Particularly, it was important to describe the prevalence in pre-school children (PSC) and women of reproductive age as they are not always targeted during mass drug administration of praziquantel. The prevalence was low in PSC using microscopy (2.3%) but reached 12.6% with PCR. For women of reproductive age, microscopic prevalence in adolescents and adults was 17.6% and 9.7%, respectively. Literature data have shown that the burden of schistosomiasis in PSC and women of reproductive age in Africa varies among countries and also regions within the same country. In Burkina Faso, a west African country, the highest prevalence seen in women was 21.3% even if young females were included [22], while in in Tanzania, only a 4.5% prevalence was reported in women [23]. Similar variations of the prevalence of PSC are also observed on the continent [24–26].

This situation is particularly interesting in the surveillance of schistosomiasis as the use of different diagnostic tools could change the choice of the control strategy or approach.

The most effective diagnostic method for the detection of *Schistosoma* in urine samples could be molecular biology. Indeed, several studies have shown the superiority of PCR techniques compared to conventional methods including the filtration technique [9, 27, 28].

In the current study, real-time PCR showed very good performance compared to the methods used in routine diagnosis. There was good sensitivity and excellent specificity. For the assessment of the infection intensity, there was good correlation between the number of eggs per 10 ml and Ct value. Median number of eggs per 10 ml was higher in the group with Ct value < 30 compared to the group with Ct value between 30 and 35. Many other studies have shown similar good correlation between egg count and Ct value. For example, Melchers et al. reported a strong correlation both before and 18 months after praziquantel treatment [29]. They also found less day-to-day variation of egg count in urine testing over 3 days. In another study, the authors gave more details on the correlation between egg count and Ct value [30]. They found that a single schistosome egg corresponded to the Ct value at 20 ± 2. Another important aspect of the PCR technique is the type of DNA extraction. Indeed, a DNA extraction procedure using a beat beating step increases the amount of *Schistosoma haematobium* DNA as reported by *Pomari *et al. [31]. For the amplification in this study, we chose the Dra 1 tandem repeat gene. This 121-pb sequence has been proposed to detect schistosomes at low level infections [20, 32]. The Dra 1 tandem repeat is used as a target gene as it is found in 15% of the entire *Schistosoma haematobium* genome. In this study, real-time PCR was able to confirm the diagnosis of schistosomiasis in patients having 1 egg/10 ml urine sample.

In summary, the real-time PCR technique is a very good diagnostic tool for the detection of *Schistosoma haematobium* in urine samples particularly in the context of the elimination of schistosomiasis. However, due to the need for a complex platform and expensive reagents, its use for public health purposes in low- and middle-income countries is limited. Therefore, it is important to do more assessments in the field on isothermal amplification technologies including LAMP and RPA techniques. They have been shown to be as sensitive and specific as real-time PCR but less expensive [33, 34].

## Conclusion

Diagnosis is an important aspect in the control and elimination of schistosomiasis as a public health problem. Microscopic methods are used and recommended by WHO as the gold standard. However, in low endemic areas, there is a need to use more sensitive tools including molecular techniques to assess the interruption of transmission. This study has shown the importance of real-time PCR technique particularly in pre-school-aged children and women of reproductive age. Further studies assessing isothermal amplification technologies in the field are needed for public health purposes.

## Data Availability

The datasets used in this study are available upon reasonable request from the corresponding author.

## Abbreviations

PSC: Pre-school-aged children
POC-CCA: Point-of-care circulating cathodic antigen
